# Attention to emotional stimuli in borderline personality disorder – a review of the influence of dissociation, self-reference, and psychotherapeutic interventions

**DOI:** 10.1186/s40479-016-0047-z

**Published:** 2016-10-04

**Authors:** Dorina Winter

**Affiliations:** Department of Psychosomatic Medicine and Psychotherapy, Central Institute of Mental Health, Medical Faculty Mannheim/Heidelberg University, PO Box 12 21 20, 68072 Mannheim, Germany

**Keywords:** Borderline personality disorder, Emotion regulation, Cognition-emotion interaction, Psychotherapy, Functional magnetic resonance imaging

## Abstract

Interactions between attention and processing of emotional stimuli shed light on both sensitivity to emotional stimuli as well as emotion dysregulation. Both of the latter processes have been proposed as central characteristics of altered emotion processing in those with borderline personality disorder (BPD). This review first summarizes the conflicting behavioural, psychophysiological and neuroimaging evidence for the hypothesis that emotional dysregulation should be reflected by higher distractibility through emotional stimuli in those with BPD. Dissociation, self-reference, as well as symptom severity modulated by psychotherapeutic interventions are proposed to help clarify divergent findings. Data suggest an association of dissociation with impaired task continuation during the presentation of interfering emotional and neutral stimuli, as well as high recruitment of neuronal attention networks together with a blunted emotional response. Considering self-reference, evidence suggests that negative rather than positive information may be more self-relevant to those with BPD. This may be due to a negative self-concept and self-evaluation. Social or trauma-relevant information attracts more attention from individuals with BPD and thus suggests higher self-relevance. After psychotherapeutic interventions, initial evidence may indicate normalization of the way attention and emotional stimuli interact in BPD. When studying attention-emotion interactions in BPD, methodological heterogeneities regarding sample, task, and stimulus characteristics need to be considered. When doing so, dissociation, self-reference, and psychotherapeutic interventions offer promising targets for future studies on attention-emotion interactions in those with BPD. This could promote a deeper insight into the affected individuals’ struggle with emotions.

## Background

**Fig. 1:**
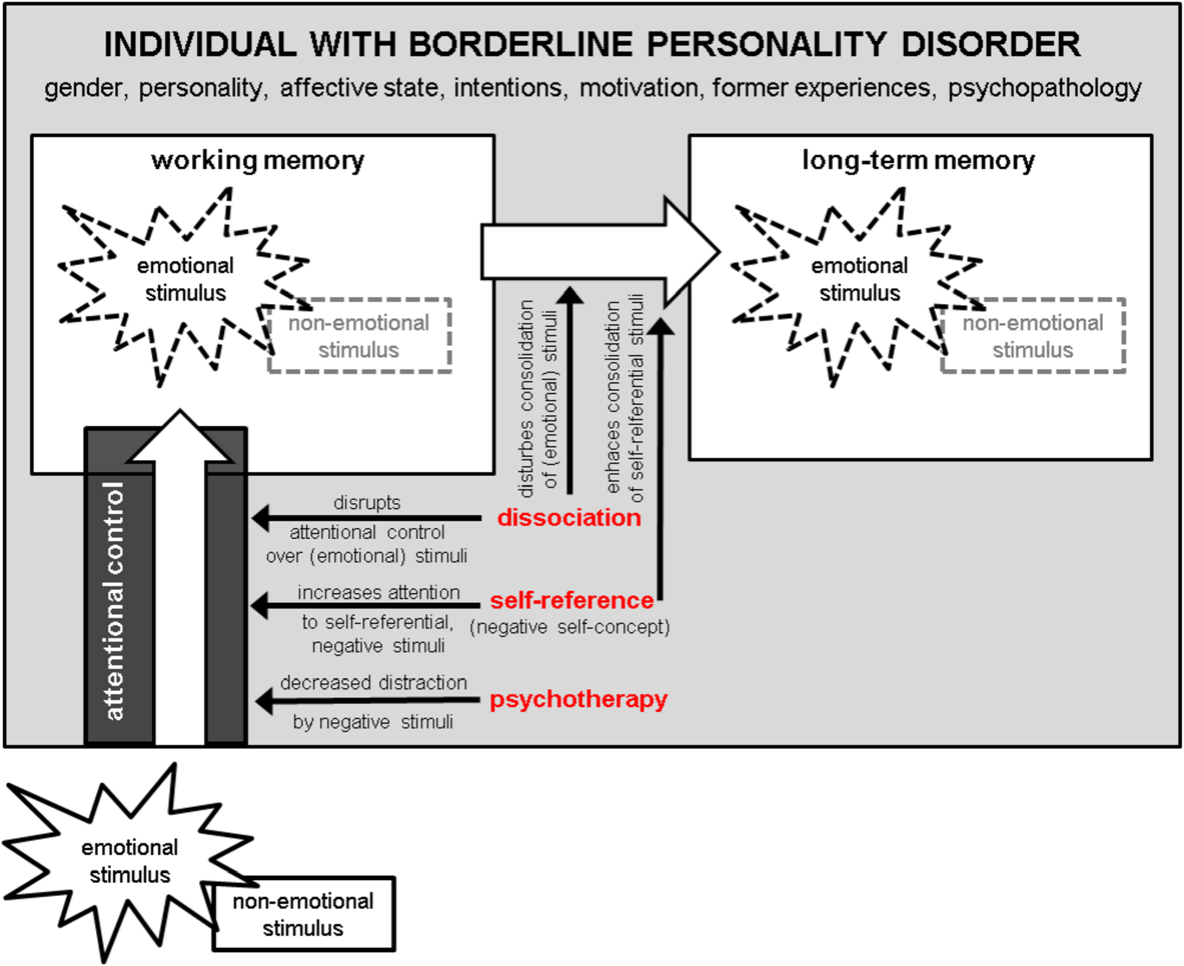
Schematic summary of the proposed influence of dissociation, self-reference and psychotherapeutic interventions on attention and memory during the processing of emotional stimuli. This figure summarizes how dissociation, self-reference and symptom severity may modulate the processing of emotional information in individuals with borderline personality disorder, starting from attention to the stimulus, encoding into working memory, storage into long-term memory and thus, again, modulating the attentional focus selected. Figure adapted from Winter et al., [195]

Emotional dysregulation constitutes the central characteristic of borderline personality disorder (BPD [1–3]). This is reflected in all three BPD core pathology domains: affective dysregulation, interpersonal disturbances and behavioural dysregulation [4, 5]. Indeed, the ability to control emotional reactions has been identified as an important capability to maintain mental health for everyone [6–8]. One can use mental capabilities to modulate emotional reactions in different ways. For example, one’s attentional focus quite efficiently modulates the processing of emotional stimuli, including emotional responses–but this is also the case vice versa (for reviews see [9–13]). These mutually influencing processes will further be called attention-emotion interactions. Attention constitutes a filter favouring e.g. emotional information, which is then preferentially stored in long-term memory and consequently influences subsequent cognitive processes and behaviours congruently (see Fig. 1) [14]. Thus, attention-emotion interactions offer information on the way a person processes emotional stimuli: First, they indicate *emotional sensitivity,* which describes how easily a person reacts to emotional information. Even in healthy populations, emotional information attracts more attention compared to less emotional information [9, 10, 15]. Sensitivity to emotional stimuli prepares and facilities selective reactions to potentially relevant environmental cues while inhibiting concurrent–and potentially distracting–stimuli and actions [16, 17]. However, this mechanism can become disadvantageous, when emotional stimuli disrupt the processing of target stimuli and goal-directed behaviours [18–23]. Second, the ability to direct attentional focus to or away from a stimulus is a common *emotion regulation* strategy, called “distraction” [24]. In certain tasks in which regulating reactions to emotional stimuli is not intentional, it also has been considered an implicit emotion regulation strategy [25]. In general, shifting attention away from an emotional stimulus is an effective and comparatively fast strategy [26, 27] to reduce negative affect [27–33] and negative cognitions [34], particularly when stimuli are highly arousing [35].

*Brain imaging studies* (for reviews see [9–11]) indicated that attention-emotion interactions involve brain regions associated with the active maintenance of goal-relevant information in working memory, for example the dorsolateral prefrontal cortex (dlPFC) and the lateral parietal cortices [36–39]. Also, higher activity in brain regions indicating emotion processing, including the amygdala, the medial prefrontal cortex, and the ventrolateral prefrontal cortex (vlPFC) was found [40–44]. In addition, higher activity in occipital and occipito-temporal brain regions during attention-emotion interactions [45–48] suggests sensory amplification [10]. The anterior cingulate cortex (ACC) has often been associated with attention-emotion interactions, but its role is inconsistent [49–53]. One distinction is between the dorsal ACC/midcingulate cortex, which may rather indicate cognitive conflict, and the rostral/perigenual and subgenual ACC, which may rather indicate emotional conflict [54–57]. More particularly, successful attentional control when processing emotional stimuli has been associated with higher activity in the inferior frontal gyrus (IFG)/vlPFC [58, 59]. On the contrary, higher emotional conflict has been associated with higher ACC and dlPFC activity [50–53, 55, 56, 60, 61].

Not surprisingly, studies examining emotion processing (predominantly viewing emotional and neutral pictures) in BPD reveal more or less the described neural networks to be disrupted in comparison to healthy control participants (for reviews see [62–64]). But what about tasks directly targeting attention-emotion interactions?

## Attention-emotion interactions in BPD–current evidence

Studying attention-emotion interactions has been of strong interest, particularly to gain insight into emotional sensitivity in those with BPD. While individuals with BPD report particularly long and intense emotional reactions [65–68], *behavioural* measures of attention-emotion interactions do not provide clear evidence. Paradigms examining emotional sensitivity mainly require participants to perform a task, e.g. letter memorizing or colour naming, during the presentation of interfering emotional content of words or photographical pictures. If reaction times are longer, more mistakes or better memory is exhibited for emotional compared to control stimuli, they are assumed to attract more attention. Previous studies partially revealed longer reaction times and/or less accuracy during the presentation of negative [69–74] and positive [70] in contrast to neutral content in those with BPD compared to healthy controls. However, a substantial number of studies have not found any group differences [75–78]. Overall, apparently, more intense stimuli [79] with personal relevance [69–71] are more likely to provide behavioural evidence for higher attention to distracting emotional stimuli in those with BPD.

*Functional neuroimaging studies* found that, in comparison to healthy controls, individuals with BPD showed higher activity in areas indicating stronger emotional processing, such as the amygdala [79, 80] and insula [79] for negative pictures in comparison to a control condition. Also, higher medial prefrontal cortex activity has been observed in BPD for task interfering positive compared to neutral stimulus content [81]. When word stimuli were used as distractors, activity was elevated in the superior temporal gyrus–a brain region associated with semantic processing [81]. In the subgenual and perigenual parts of the ACC and adjacent brain areas, the difference between negative and neutral distracting stimuli was smaller in the BPD group than in the healthy control group [78, 82, 83]. In the dorsal ACC, similarly, some studies found a smaller activity difference for negative compared to neutral stimuli, while one study reported higher activity for positive vs. neutral stimuli in BPD [78, 81, 83]. Additionally, individuals with BPD show stronger coupling between the amygdala as well as the dACC with brain regions indicating (among other) increased stimulus saliency (medial prefrontal cortex/hippocampus for the amygdala or medial prefrontal cortex/insula/posterior cingulate/frontoparietal cortices for the dACC), during the processing of aversive distractors [84]. Taken together, referring to the role of these noted brain areas in attention-emotion interactions [9–11], these data suggest that task-irrelevant emotional information has particular salience for individuals with BPD. Concurrently, findings on a smaller ACC activity difference as response to negative compared to neutral stimuli could also indicate a smaller difference in the emotional conflict elicited by negative compared to neutral stimuli. This finding is supported by previous evidence suggesting more negative evaluations of information previously rated as neutral by healthy control samples [85, 86].

In *summary*, evidence suggests that individuals with BPD tend to show an enhanced distractibility by emotional content than healthy controls and stronger processing of the distracting emotional stimuli, suggesting higher sensitivity to emotional stimuli. However, evidence is somewhat heterogeneous on the behavioural level as well as with respect to the involvement of specific brain regions. This heterogeneity may be due to methodological differences such as sample or task characteristics ([71, 75, 76]; see also below “Consideration of Methodological Heterogeneities”). Also psychological mechanisms have been suggested to further modulate attention-emotion interactions in those with BPD [69–71, 75, 76, 79, 81, 84, 87–90]. Three important, but understudied, processes are: dissociation [79, 81, 84, 88–90], self-reference of stimulus content [69–71], and psychotherapeutic interventions [87].

## Dissociation

Dissociation is defined as the “disruption of and/or discontinuity in the normal integration of consciousness, memory, identity, emotion, perception, body representation, motor control, and behavior” [91, 92]. As such, dissociation is a subjective experience, which is usually assessed via self-reported symptom intensities. The vast majority of individuals with BPD experiences transient dissociative symptoms [93], particularly when stress levels are high [68, 93, 94]. Even though it is a salient phenomenon in BPD, the influence of dissociation on attention-emotion interactions is not well understood. Individuals with BPD experiencing recurrent dissociation showed broad deficits in neuropsychological *behavioural* and *psychophysiological* measures, including attention, executive functioning, working memory, long-term memory, and general cognitive abilities, while BPD patients who rarely experience dissociation only showed deficits in executive functioning [88]. High levels of current dissociation were also associated with an impaired ability to maintain task-related performance when facing distracting information [81]. With respect to general processing of negative stimuli, individuals with BPD reported lower pain sensitivity [95, 96] and worse aversive conditioning [97, 98] during high levels of dissociation. Furthermore, high levels of dissociation mediated startle responses and skin conductance in response to negative pictures [89]. Although not always addressing attention-emotion interactions themselves, these studies would suggest impaired attentional control on the one hand, as well as diminished sensitivity to emotional stimuli on the other hand when levels of dissociation are high in BPD.

With respect to *brain activity correlates*, higher dissociation levels in those with BPD were associated with lower amygdala activity in response to repeatedly presented negative pictures [99]. More specific to attention-emotion interactions, high dissociation levels in those with BPD correlated with reduced amygdala (and hippocampal) activation [79]. In addition, a stronger coupling between the amygdala and the ACC was found in BPD individuals with high levels of dissociation during the presentation of distracting negative stimuli [84]. Induced dissociation during a colour-naming task was also associated with higher IFG activity during distracting negative compared to neutral stimuli [81]. These findings support current models of dissociation in post-traumatic stress disorder (PTSD) [100, 101] and depersonalization disorder [102], highlighting their trans-diagnostic relevance. Both models hypothesize blunted reactivity to negative stimuli in areas associated with emotion processing (i.e., amygdala), as well as higher activity in areas associated with processing emotional conflict and emotion regulation (ACC, medial and dlPFC) in these patient groups during the processing of emotional stimuli. Such a brain pattern was previously summarized as “emotional overmodulation” [100, 101], which fits well with the above-mentioned behavioural and psychophysiological evidence. Potential emotional reactions may be excessively regulated, leading to reduced emotional responses. Unfortunately, the reported study designs do not allow answering the question whether of a “normalization” of brain activity occurring in BPD during dissociation can be observed in comparison to healthy participants e.g. in limbic brain regions. However, it does suggests that additional aberrant activity within–and connectivity to–brain regions associated with emotion regulation could be expected during dissociation in those with BPD. This would oppose the idea of an overall normalization of neural activity during dissociation.

In *summary*, few studies examined the direct modulation of attention-emotion interactions by dissociation in those with BPD [79, 81, 84]. However, the overall evidence supports two main findings for those with BPD: 1) maintaining attention to a target task during the presentation of interfering emotional and neutral information is impaired during dissociation, and 2) attention-emotion interactions during dissociative states may be characterized by high recruitment of attentional (and emotion regulation) resources associated with a blunted emotional response. This suggests dysfunctional allocation of attentional resources during attention-emotion interactions, namely for emotion regulation rather than task performance. Future studies need to clarify further the role of dissociation as trait [88, 99] versus state [79, 81, 84, 89, 95–98], as it appears that this distinction may be relevant [81].

## Self-reference

It has been argued that emotional cues are processed preferentially as they may contain information relevant to oneself, allowing goal-directed behaviours [16, 17]. This notion is supported by the fact that a person’s personality traits, state, as well as intention, motivation and decision making processes are known to influence attention-emotion interactions, in terms of so-called “top-down modulations” [103–106]. However, in favour of experimental control, most studies use standardized emotional stimuli, such as words and/or pictures. Nevertheless, this neglects the extent to which this information may refer to the individual participants. For example, participants with BPD evaluated positive or neutral words more negatively than healthy participants when these were presented with reference to themselves rather than with reference to another person [85]. In studies already mentioned on attention-emotion interactions in BPD, emotional stimuli attracted particular attention when the stimuli were selected individually [71] or when referring to BPD-related issues [69, 70].

Although several studies suggest alterations in *neural networks* associated with self-referential processing in a resting state or during self-referential processing [90, 107–109], evidence on the role of self-reference for attention-emotion interactions in BPD is sparse. In one case, an intensifying role of self-relevance on activity differences between a group with BPD and healthy participants e.g. in the IFG and the rostral ACC can be inferred [78]. One EEG study using a self-referential encoding task also reported a more intensive response to negative compared to positive traits in an event-related potential which has been associated with enhanced stimulus salience, in youth with BPD, in comparison to healthy participants [110]. However, both studies lack direct comparison between self- vs. non-self-referential (emotional) information, limiting conclusions regarding the role of self-reference on attention-emotion interactions in individuals with BPD.

If self-reference matters, which *type of stimulus content* may elicit particularly strong self-reference in those with BPD? As demonstrated through the preferential processing of positive, self-related stimuli in the general population [111–115], a person’s self-concept appears to be highly relevant. Individuals with BPD report low self-esteem [116, 117] and provide negative self-descriptions [118]. They show high levels of self-criticism and feelings of inferiority [119] and are more shame-prone [120]. They also consider their body as particularly aversive [121, 122]. In light of this evidence and supported by a study in youth with BPD [110], negative stimuli likely provide more self-reference and positive stimuli provide less self-reference in those with BPD, thus, differentially affecting attention-emotion interactions and probably its neural correlates.

Additionally, self-descriptions and attitudes towards themselves are at least somehow *unstable* in those with BPD [92, 107, 123, 124]. This is in line with the experience of a poor sense of self, which constitutes a diagnostic criterion of BPD [92]. Accordingly, it is reasonable to assume that there could be certain instabilities in the way one refers to stimuli to oneself [125, 126]. However, such instabilities have not yet been addressed with respect to attention-emotion interactions.

Finally, stimuli used to study attention-emotion interactions also involve events, photographic scenes, and/or faces. Due to the interpersonal difficulties frequently experienced by individuals with BPD, social information may be of reasonably higher self-relevance and thus attract more attention, as supported by a recent study [74]. In line with this, a trend to higher attention to self- in comparison to other-referential, social information has been suggested by a social memory paradigm [86]. Further, a majority of individuals with BPD report significant, repetitive aversive childhood experiences [127–129] and PTSD is also frequently diagnosed in those with BPD (17–39.2 %; [130–132]). Thus, trauma-related stimuli may attract more attention in BPD than in healthy controls.

In *summary*, individuals with BPD show a marked negative self-concept and low self-esteem, which may be unstable. Social or trauma-relevant information may provide particular self-reference for those with BPD. Thus, negative, social, or trauma-relevant information may lead to stronger attention-emotion interactions in those with BPD than in control groups, namely by capturing more attention as well as additional recruitment of neural networks involved in self-referential processing (e.g. cortical midline structures [133]).

## Psychotherapeutic interventions and symptom severity

Psychotherapeutic interventions suggest medium effect sizes with respect to symptom reduction in BPD [134–136] but related alterations in attention-emotion interactions have been seldom examined [87, 137, 138]. Such changes could be indicators of both improved emotional sensitivity as well as emotion regulation.

On the *behavioural level*, there is one study reporting prolonged reaction times to BPD-relevant stimulus content normalized in those who recovered from BPD after 3 years of outpatient psychotherapy [87]. Neuroimaging studies, however, did not find significant behavioural alterations post therapy [137, 138].

Which changes in *neural correlates* of attention-emotion interactions can be expected after psychotherapy? Reviews have suggested that altered brain patterns normalized after psychotherapy in other mental illnesses such as obsessive-compulsive disorder, depression, and schizophrenia [139–141]. However, there may be an increased level of activity in additional brain areas after psychotherapy, as observed in panic disorder and PTSD [140–142], which may indicate a ‘compensatory’ brain response obtained to cope with challenging stimuli and responses. Studies focusing on neural correlates of emotion processing in BPD patients found that activity of brain regions associated with emotion processing (i.e., the amygdala) declined after successful psychotherapy targeting emotion regulation [143–145]. With respect to attention-emotion interactions, a pre-post design study also showed increased dorsal prefrontal (dorsal ACC, dlPFC, frontopolar cortex) and decreased vlPFC and hippocampal activity, while BPD participants viewed distracting negative stimuli from before to after transference-focused psychotherapy [138]. This suggests increased response in brain areas that control attention to (emotional) stimuli and decreased response in brain regions associated with emotional response. However, due to the lack of a respective control group in this study, it is not possible to conclude whether this represents a normalization of brain activity patterns. A study by our research group found decreased activity over time in the inferior parietal lobe/supramarginal gyrus during distraction from negative rather than neutral stimuli in those with BPD from before to after dialectical behaviour therapy (DBT), which was correlated with improvements in self-reported borderline symptom severity [137]. Thus, in future studies, both a normalization of fronto-parietal-limbic activity as well as additional recruitment of frontal areas involved in the executive system could be expected in response to distracting emotional stimuli following symptom improvement in BPD after psychotherapy.

Overall, evidence for alterations of alterations of attention-emotion interactions after psychotherapy in BPD is still very preliminary and offers many future research directions. Over the last few decades, several psychotherapeutic approaches have been developed or adapted specifically to treat individuals with BPD [135]. However, only DBT and transference-focused psychotherapy explicitly studied attention-emotion interactions in those with BPD [137, 138]. Here, specific psychotherapies may go in hand with specific alterations in attention-emotion interactions. For example, DBT, which focuses on emotion regulation [146], may be associated with a decrease in sensitivity to emotional stimuli and an increased ability to regulate emotional responses. In contrast, schema-focused therapy [147], which focuses on strong emotional reaction to self-relevant triggers, may be associated with normalized sensitivity to self-relevant emotional triggers and thus, less effort to control attention over triggers. It would be optimal if future randomized controlled trials included paradigms to measure different types of attention-emotion interactions and their neural correlates, in order to gain knowledge regarding intervention-specific alterations in BPD.

In addition to psychotherapy, future research on attention-emotion interactions could also examine non-psychotherapeutic interventions for those with BPD. For example, specific neural targets such as the amygdala, insula, ACC, IFG/vlPFC or dlPFC can be trained in those with BPD using functional magnetic resonance imaging (fMRI) feedback – referred to as real-time fMRI neuro-feedback [148–151] – allowing for attention-emotion interactions and their neural correlates to be modulated. Brain stimulating techniques (e.g., transcranial magnetic stimulation or transcranial direct current stimulation) may be of interest, as they are known to modulate attention-emotion interactions [152–154]. Additionally, pharmacological interventions, which target the oxytocin system [155, 156] or hypothalamic-pituitary-adrenal axis functioning [157], have revealed enhanced attentional control of/reduced interference by negative stimuli in those with BPD. Finally, antidepressants may also alter attention-emotion interactions in BPD patients [158–160].

In *summary*, even though initial evidence suggests normalization of attention-emotion interactions following psychotherapeutic interventions, future research needs to add to this evidence and to clarify the role of specific interventions regarding these processes.

## Consideration of methodological heterogeneities

Per se, BPD is a very heterogeneous mental condition, with five out of nine criteria to be met to obtain the diagnosis [92]. In extreme cases, two individuals with BPD may fulfil only one joint BPD criterion. To account for this, it may be relevant to either select or – even better – compare individuals with BPD who show certain symptoms of interest (e.g. [81, 137]) or to link results to symptom domains (e.g. [79, 138]). Apart from these BPD eminent heterogeneities, the reported studies show methodological heterogeneities in sample and task characteristics, which need to be considered when interpreting their findings.

With respect to *sample characteristics*, the role of comorbid disorders in the BPD samples needs further exploration. Evidence suggests that PTSD, dissociative disorders, major depression and attention deficit hyperactivity disorder may influence emotion processing and attention-emotion interactions in those with BPD [71, 161–165].

In addition, individual arousal, mood, or emotional state has been shown to influence attention-emotion interactions state-congruently [166–168]. As individuals with BPD experience more intense and prolonged emotional states compared to healthy controls [65–68], it is likely that their emotional states influence attention-emotion interactions more differentially than observed in healthy controls.

Further, findings cannot be generalized across gender, as many studies predominantly included women (except [80]). This is relevant, as gender influences emotion processing and regulation [169–171]. In BPD research, further gender differences can be expected, as men often show lower overall symptomatology compared to women [172, 173] and differ in their comorbidity profile (i.e., males: more often antisocial personality disorder; women: more often PTSD and eating disorders [131, 172–175]). Also, serotonergic functions, which have been associated with emotion processing and impulsivity, appear to differ between genders in those with BPD [176–178]. Thus, it is reasonable to study male and female individuals with BPD separately, but also comparatively.

When interpreting results, it is necessary to consider that many studies did not exclude the use of psychotropic medication, even though they are known to alter emotion processing and its neural correlates [62, 179–182]. It has been proposed to include medication load as a covariate in analyses, if the statistical design and the variance in medication provides enough power to do so [64]. Future research may also examine the influence of different types of medication on attention-emotion interactions in BPD.

In *summary*, several sample characteristics such as comorbidities, emotional state, medication and gender may influence a study’s results and therefore may be of interest for its interpretation. Often, due to comparatively small sample sizes and thus, power challenges, analyses addressing these issues are omitted or cannot be conducted to a considerable degree. Thus, examining the role of these variables on attention-emotion interactions in BPD need to be subject to future research.

With respect to *task characteristics*, it needs to be stressed that this review aggregated findings across different task types, as the amount of available studies was mainly limited. The tasks used in the reported studies have been discussed to examine specific mental processes, such as response inhibition and impulse control (for further information see [161, 183]). These tasks also differed in their difficulty level, e.g. through (slower or faster) timing or (lower or higher) cognitive load. In the general population, if the task has low to moderate difficulty, higher difficulty is associated with stronger attention-emotion interactions as evinced by prolonged reactions in the presence of distracting emotional compared to neutral stimuli [184–186]. However, if task difficulty is very high (e.g. as evinced by many mistakes made), this effect is not present anymore [10, 187–189]. With respect to BPD, this would suggest that altered attention-emotion interactions may be observed most likely at moderate to difficult tasks (e.g. emotional working memory task [79]), while no or smaller effects may be observed when with easy or very difficult tasks (e.g. emotional Stroop task [78, 81]).

Furthermore, *stimulus characteristics* need to be considered, as stimulus types such as faces and scenes are associated with stronger differences in task performance during the presentation of emotional compared to neutral stimuli than words [9, 190]. In addition, studies often include stimuli that are exclusively of negative or rather neutral valence. Positive items have rarely been used [70, 85], limiting this review of attention-emotion interactions mainly to negative stimuli. Particularly for BPD, it is often debated whether neutral items are actually neutral or rather negatively biased [63, 64, 191], questioning the usual procedure to contrast emotional stimuli with neutral stimuli [69–73, 75–78, 81]. However, it is under debate as to which would be the optimal comparison condition. It has been suggested to directly compare negative and positive stimuli [64]. However, participants rated negative stimuli as more arousing than positive ones, even when the authors had matched negative and positive stimuli according to standardized ratings of this dimension [192]. Thus, in these cases, the confounder remained that stimuli of higher arousal attract more attention [193, 194]. With respect to brain imaging studies, it also supervenes that specific pathways for valence and arousal dimensions could hardly be identified [9]. A suggestion for future research could be to include positive stimuli and report and control for valence and arousal ratings of the stimuli rated after participation by the participants.

In *summary*, the methodological heterogeneities in the reported and additional studies suggest that sample characteristics, task and stimulus characteristics may bias attention-emotion interactions. Well-powered and well-controlled study designs may be able to examine the influence of these variables in future studies.

## Conclusions

This review first provided an overview of the concept and neural correlates of attention-emotion interactions in the general population. Heterogeneous evidence was presented to demonstrate that it is not yet clear whether emotional dysregulation in those with BPD is reflected by higher distractibility by emotional stimuli. This review then summarized evidence for three selected, not yet well-studied, factors (i.e., dissociation, self-reference, and psychotherapeutic interventions), which are suggested to modulate attention-emotion interactions in those with BPD. They thus, in future, may allow a clearer understanding of the mechanisms influencing attention-emotion interactions in those with BPD. Figure 1 gives an overview of the main findings on attention-emotion interactions and dissociation, self-reference, and psychotherapeutic interventions. Dissociation was found to be associated with impaired maintenance of task continuation during the presentation of interfering emotional and neutral information in those with BPD, as well as high recruitment of attentional resources associated with a blunted emotional response. With respect to self-reference, this review points out that individuals with BPD show a marked negative self-concept and low self-esteem, which may be unnstable, and increase attention-emotion interactions for negative stimuli. Social or trauma-relevant information may also provide more self-reference for individuals with BPD compared to healthy controls. After psychotherapeutic interventions, initial evidence suggests normalization of attention-emotion interactions. All of these processes not only influence attention-emotion interactions, but also influence how information is subsequently stored in long-term memory [14, 195], leading to biased representation of the outside world and, again, influencing attention-emotion interactions. As evidence is often heterogeneous, the proposed influences need to be considered as working hypotheses for future research. In this research, several methodological challenges regarding sample, task, and stimulus characteristics need to be considered. If done so, dissociation, self-reference and symptom improvement via therapy may offer promising targets to understand the conflicting evidence found in attention-emotion interactions in those with BPD. This shall increase knowledge on the mechanisms of sensitivity to emotional stimuli as well as emotion regulation in BPD.

## Abbreviations

ACC: Anterior cingulate cortex
BPD: Borderline personality disorder
DBT: Dialectical behaviour therapy
dlPFC: Dorsolateral prefrontal cortex
e.g.: exempli gratia; *for example*
fMRI: functional magnetic resonance imaging
i.e.: id est; *that is*
IFG: Inferior frontal gyrus
PTSD: Post-traumatic stress disorder
vlPFC: Ventrolateral prefrontal cortex
